# A reporter cell line for the automated quantification of SARS-CoV-2 infection in living cells

**DOI:** 10.3389/fmicb.2022.1031204

**Published:** 2022-09-29

**Authors:** Lowiese Desmarets, Nathalie Callens, Eik Hoffmann, Adeline Danneels, Muriel Lavie, Cyril Couturier, Jean Dubuisson, Sandrine Belouzard, Yves Rouillé

**Affiliations:** ^1^CNRS UMR 9017, INSERM U1019 Centre d’Infection et Immunité de Lille (CIIL), Institut Pasteur de Lille, Université de Lille, Lille, France; ^2^INSERM U1177-Drugs and Molecules for Living Systems, Institut Pasteur Lille, Université de Lille, Lille, France

**Keywords:** coronavirus, SARS-CoV-2, antivirals, high content imaging, screening, 3CL^pro^, nsp5, M^pro^

## Abstract

The SARS-CoV-2 pandemic and the urgent need for massive antiviral testing highlighted the lack of a good cell-based assay that allowed for a fast, automated screening of antivirals in high-throughput content with minimal handling requirements in a BSL-3 environment. The present paper describes the construction of a green fluorescent substrate that, upon cleavage by the SARS-CoV-2 main protease, re-localizes from the cytoplasm in non-infected cells to the nucleus in infected cells. The construction was stably expressed, together with a red fluorescent nuclear marker, in a highly susceptible clone derived from Vero-81 cells. With this fluorescent reporter cell line, named F1G-red, SARS-CoV-2 infection can be scored automatically in living cells by comparing the patterns of green and red fluorescence signals acquired by automated confocal microscopy in a 384-well plate format. We show the F1G-red system is sensitive to several SARS-CoV-2 variants of concern and that it can be used to assess antiviral activities of compounds in dose–response experiments. This high-throughput system will provide a reliable tool for antiviral screening against SARS-CoV-2.

## Introduction

The severe acute respiratory syndrome coronavirus 2 (SARS-CoV-2), the etiological agent of COVID-19, was first detected at the end of 2019 (Zhu et al., 2020), after which it rapidly spread throughout the human population and resulted in a global pandemic. The rather fast production of effective vaccines soon brought hope for pandemic control. However, difficulties with vaccination strategies, the successive emergence of new and increasingly contagious variants of SARS-CoV-2, and the waning immunity conferred by these vaccines have led to the idea that effective antivirals will additionally be required to control this pandemic and protect high-risk patients. Given the urgent need for directly applicable medicines, most studies focused on repurposing drugs yet approved for other diseases (Jeon et al., 2020; Riva et al., 2020; Bakowski et al., 2021; Belouzard et al., 2022). With time, it became clear that some broad-spectrum antiviral molecules, especially targeting the RdRP of a wide range of RNA viruses (such as remdesivir, favipiravir, and molnupiravir) also showed a protective activity against SARS-CoV-2 *in vitro* and in animal models (Kaptein et al., 2020; Shannon et al., 2020; Sheahan et al., 2020; Abdelnabi et al., 2021) and some of them have now been tested in clinical studies and approved for use in (high-risk) patients.

Apart from the RdRP, the search for more specific SARS-CoV-2 antivirals has focused on the SARS-CoV-2 main protease, known as nsp5, 3CL^pro^, or M^pro^, this is in line with earlier studies that successfully identified 3CL^pro^ inhibitors against other CoVs, such as PF-00835231 for SARS-CoV-1 (Hoffman et al., 2020) and GC376 for feline CoVs (Kim et al., 2012, 2013, 2015), both of which also show anti-SARS-CoV-2 antiviral activity (Fu et al., 2020; Ma et al., 2020; Vuong et al., 2020; de Vries et al., 2021). The coronaviral M^pro^ is essential for polyprotein cleavage and release of the polymerase subunits in early replication stages, and an attractive target for SARS-CoV-2 inhibition due to its unique substrate preference (Leu-Gln↓{Ser, Ala, Gly}), not used by any known human protease (Zhang et al., 2020b). The identification of additional and specific SARS-CoV-2 M^pro^ inhibitors has mainly been approached by structure-based design, crystallographic analyses, FRET-based substrate cleavage, and other biological screening assays (Dai et al., 2020; Hoffman et al., 2020; Jin et al., 2020; Zhang et al., 2020a,b, 2021; Ma et al., 2021; Kitamura et al., 2022; Quan et al., 2022; Unoh et al., 2022). Subsequent cell-based infection assays and/or animal experiments revealed high potency of several compounds (Zhang et al., 2020b; Owen et al., 2021; Abdelnabi et al., 2022; Quan et al., 2022; Unoh et al., 2022), of which one (PF-07321332/Nirmatrelvir) has been approved for clinical use so far.

Simple and efficient cell-based assays have not only been essential to confirm the antiviral properties of *in silico* designed inhibitors, but have been equally indispensable for broader antiviral screenings and hence for finding other antiviral targets, as exemplified by the discovery of potent antivirals targeting non-enzymatic viral proteins, such as HCV NS5A, which are now being used to treat HCV-infected patients (Gao et al., 2010), or a highly potent dengue virus inhibitor targeting the NS3-NS4B interaction (Bardiot et al., 2018; Kaptein et al., 2021). Besides the classical low-throughput plaque reduction and RT-qPCR assays, potential high-throughput cell-based methods for SARS-CoV-2 antiviral screening include assays based on the use of recombinant infectious viruses or replicons expressing a luciferase and/or fluorescent protein (Xie et al., 2020; Ricardo-Lax et al., 2021; Tanaka et al., 2022), which, however, are not easily adaptable to the multiple and quickly evolving SARS-CoV-2 variants. Other assays not restricted to a single variant are based on immunofluorescence staining using antibodies against specific SARS-CoV-2 proteins (Bakowski et al., 2021) or on the assessment of the general cytopathic effect or of syncytia formation several days after inoculation (Buchrieser et al., 2020; Riva et al., 2020; Belouzard et al., 2022; Chiu et al., 2022), but those approaches are often quite time-consuming and/or labor-intensive. In order to develop an easier-to-use assay, the aim of this study was to construct a fluorescent reporter cell line, allowing for a fast and automated read-out of the SARS-CoV-2 infection, and requiring minimal handlings to ensure maximum safety in a BSL-3 environment. In this study, we describe the construction and optimization of this reporter approach and its adaptation to phenotypic high-content confocal imaging to monitor SARS-CoV-2 infection.

## Materials and methods

### Chemicals

DMEM, fetal bovine sera, and DAPI were purchased from Life Technologies. Chloroquine and GC376 were from Sigma. Remdesivir and molnupiravir were from Bio-Techne and Euromedex, respectively.

### Cells

Vero-81 cells (ATCC number CCL-81) and HEK-293 T cells were maintained in DMEM supplemented with 2 mM GlutaMax and 10% fetal bovine serum. HeLa cells were maintained in minimum essential medium α supplemented with 2 mM GlutaMax and 10% fetal bovine serum.

### Viruses

SARS-CoV-2 variants including the original Wuhan strain (EPI_ISL_410720, kindly provided by the French National Reference Center for Respiratory Viruses hosted by Institut Pasteur (Paris, France)), the strain containing the D614G mutation (EPI_ISL_940555), the alpha (B1.1.7; EPI_ISL_1653931), the Beta (B.1.351; EPI_ISL_1653932), the Delta (B.1.617.2; EPI_ISL_7696531), and Omicron (EPI_ISL_7696645) variants were all propagated in Vero-E6 cells expressing TMPRSS2.

A recombinant Sindbis virus (SINV) expressing HCV E1 glycoprotein was employed as previously described (Ferlin et al., 2018). Yellow fever virus (YFV) strain 17D and coxsackievirus B4 strain E2 (CVB4) were kindly provided by Philippe Desprès (Institut Pasteur de Paris, France) and Didier Hober (Université de Lille, France), respectively.

### Antibodies

Mouse anti-dsRNA mAb (clone J2) was obtained from SCICONS. Polyclonal rabbit anti-SARS-CoV-2 nucleocapsid antibody was from Novus. Mouse anti-HCV E1 mAb A4 (Dubuisson et al., 1994) was produced *in vitro*. Mouse anti-GFP mAb was from Roche. Rabbit anti-calnexin antibody was from Abcam. Rabbit anti-ACE2 antibody was from Cell Signaling Technology. Mouse anti-transferrin receptor mAb was from SantaCruz. Cyanine 3-conjugated goat anti-mouse IgG and horseradish peroxidase (HRP)-conjugated goat anti-rabbit and goat anti-mouse IgG antibodies were from Jackson ImmunoResearch.

### Plasmids

To construct the fluorescent reporter, oligonucleotides 5′-AGCTTCACCAAAAAAAAAAAGAAAAGTAGGAGG-3′ and 5′-GATCCCTCCTACTTTTCTTTTTTTTTTTGGTGA-3′ containing the coding sequence of the nuclear localization signal of SV40 T antigen were phosphorylated, annealed and inserted by ligation between the HindIII and BamHI sites of the plasmid pEGFP-C1 (Promega), yielding pEGFP-NLS. The coding sequence of the cleavage site fused to that of cytochrome 5b C-terminus was amplified by PCR with primers 5′-TTTGGGATCCCCAGCGCTGTGCTGCAGAGCGGATTCCCTCCGGAAACTCTTATCACTAC-3′ and 5′-TTTGTCTAGATCAGTCCTCTGCCATGTAATCC-3′ and cDNA obtained by reverse transcription of Huh-7 cells total RNA as a template. The PCR product was purified and inserted between the BamHI and XbaI sites of pEGFP-NLS.

A plasmid expressing a similar construct with no cleavage site was constructed by inserting a PCR product obtained with a sense primer without cleavage site coding sequence (5′-TTTGGGATCCCTCCGGAAACTCTTATCACTAC-3′) and the same antisense primer. Plasmids expressing mutants LR and QN were also constructed by PCR with sense primers 5′-TTTGGGATCCCCAGCGCTGTGCGGCAGAGCGGATTCCCT-3′ and 5′-TTTGGGATCCCCAGCGCTGTGCTGAATAGCGGATTCCCT-3′, respectively. All plasmids were verified by sequencing.

The wild-type nsp5 sequence from GenBank accession MN908947.3 was codon optimized for mammalian cell expression and purchased from GeneCust. The nsp5 coding region was inserted in the pcDNA3.1(+) NheI/XhoI sites. An inactive version of nsp5 containing the mutations H41A and C145A was obtained by PCR overlap mutagenesis. The nsp5 coding sequence and flanking regions of these vectors were sequence verified.

To construct the pTRIP-myc-ACE2 vector, the coding sequence of myc-ACE2 was amplified by PCR using the pCDNA3.1-ACE2 kindly provided by Gary Whittaker (Cornell University, United States) as a template and the primers: 5′-CCGACTCTAGACCATGTCAAGCTCTT-3′ and 5′-ATATAGCTCGAGTTAAGCGGGCGCCACCTGGGA-3′. The PCR product was ligated into the pTRIP vector after restriction with XbaI and XhoI.

### Transfection of fluorescent reporters

Vero-81-derived cells were transfected using the TransIT-LT1 transfection agent (Mirus Bio). Transfected cells were selected with geneticin (2 mg/ml) for 2 weeks after transfection and GFP-expressing cells were enriched using a fluorescence-activated cell sorter. A cell line stably expressing the fluorescent reporter (F1G) was obtained by three successive limiting dilutions. Transient transfection of HeLa cells was obtained by using the same agent without subsequent selection.

### Lentivirus

A plasmid encoding an mCherry-NLS protein was constructed similar to its GFP counterpart and the coding sequence of this construct was inserted in the pRRL.sin.cPPT.SFFV/IRES-puro.WPRE plasmid (Doyle et al., 2018), kindly provided by Caroline Goujon (Institut de Recherche en Infectiologie de Montpellier, France). A lentiviral vector stock was obtained by co-transfection of HEK-293 T cells with the resulting plasmid (pRRL.sin.cPPT.SFFV/mCherry-NLS.IRES-puro.WPRE) and plasmids expressing HIV Gag-Pol and VSV-G at a ratio of 5:4:1. The culture medium was collected after 3 days at 33°C, filtered and used to transduce cells stably expressing the fluorescent reporter probe. Cells expressing the mCherry-NLS construct were selected with puromycin (10 μg/ml) followed by a limiting dilution. This yielded the F1G-Red cell line.

### Immunoblotting

Cells were lysed and analyzed by immunoblotting as previously reported (Lebsir et al., 2019).

### Immunofluorescence

Cells grown on glass coverslips were fixed and processed for immunofluorescence staining and imaged using an EVOS M5000 imaging system, as reported previously (Eymieux et al., 2021). For colocalization assessment, images were acquired using a confocal microscope (Zeiss LSM 880) using a 63x oil-immersion objective.

### High content imaging for antiviral assessment

F1G-red cells were cultured in 30 μl of complete medium for 24 h in 384-well plates (Greiner, Ref. 781091; 4,500 cells/well) and transferred to the BSL-3 facility. 30 μl of antiviral solutions in three-fold dilution series was added, after which the cells were inoculated with 10 μl of SARS-CoV-2 (MOI of 0.2) in a complete medium to obtain a final antiviral drug concentration ranging from ~0.1–45 μM. Cells were incubated for 16–18 h at 37°C and 5% CO_2_ followed by image acquisition using an InCell-6500 automated confocal microscope (Cytiva) equipped with a 20x CFI Plan APO objective (NA: 0.75). Nine fields per well were analyzed using the 488 nm and 561 nm laser lines adjusted to 90% laser power for excitation of the GFP and mCherry channels, respectively, and applying a 4-band emission polychoric filter (GFP: 500–550 nm, mCherry: 570–630 nm). Aperture values were set at 2.85 and technical triplicates were acquired for each experimental condition. In each experiment, identical sets of antiviral dilutions were applied to non-infected cells to control for the lack of impact of the compounds on the intracellular localization of GFP. Percentages of infection were measured by automated image analysis using the Columbus software (version 2.9.1, PerkinElmer) and an adapted script to distinguish between infected and non-infected cells (Supplementary Table 1). Data were analyzed using the nonlinear fit of log-dose vs. response function of GraphPad Prism (version 6) to calculate EC_50_ values. The total number of cells was also measured and analyzed similarly using mCherry signals to assess the toxicity of the compound. The Z’-factor was calculated using the formula 1–3*(MAD(positive control) + MAD(negative control))/abs(median(positive control)-median(negative control)), MAD being the median absolute deviation.

## Results

### Isolation of Vero-81 cell clones with increased susceptibility to SARS-CoV-2

**Figure 1:**
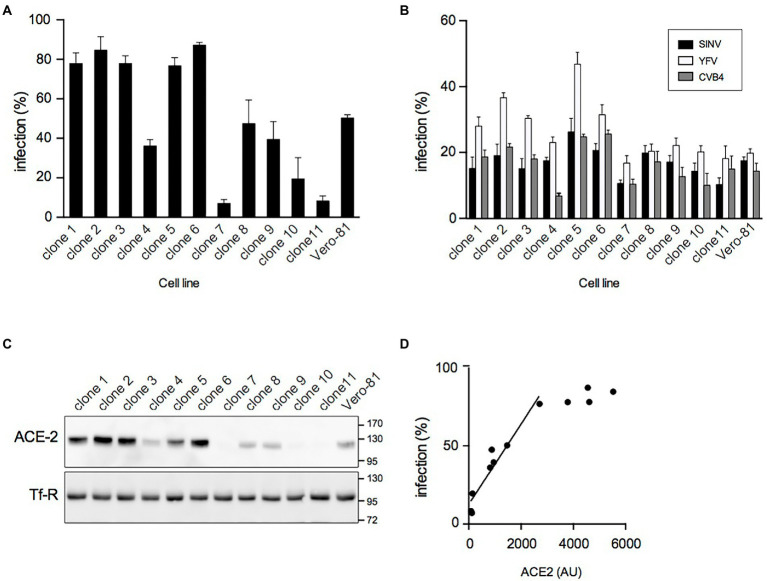
Isolation of highly susceptible Vero-81-derived cells. **(A)** Clonal cell lines were derived from Vero-81 by limiting dilution cloning and infected with SARS-CoV-2. The percentage of infection was measured with an immunofluorescence assay to dsRNA (mean ± SEM of 3 independent experiments). **(B)** Isolated clones and parental Vero-81 were infected with Sindbis virus (SINV), yellow fever virus (YFV), or coxsackievirus B4 (CVB4). The percentage of infection was measured with immunofluorescence assays to dsRNA (YVF and CVB4) or to E1 (SIN) (mean ± SD of 6 replicates). **(C)** Immunoblot analyses of ACE2 and transferrin receptor (Tf-R) expression in isolated clones and parental Vero-81. **(D)** Scatter plot showing the percentage of infection by SARS-CoV-2 as a function of ACE2 expression and the linear regression curve obtained for the clones with lower ACE2 expression levels. AU, arbitrary unit.

Previous experiments showed that the number of SARS-CoV-2 infected Vero-81 cells reached a plateau of about 70% at 24 hpi independent of the used MOI (Eymieux et al., 2021). As this might result from the presence of resistant cells in the Vero-81 cell population, individual clones were isolated by limited dilution cloning, and their susceptibility to SARS-CoV-2 was analyzed. Large variations in infection levels, ranging from 7% to 87%, were observed between clones (Figure 1A), confirming the presence of a mixture of resistant and susceptible cells in the original population.

To further characterize these clones, their susceptibility to infection by three other (+)RNA viruses, namely coxsackievirus B4 (CVB4, family *Picornaviridae*), yellow fever virus (YFV, family *Flaviviridae*), and Sindbis virus (SINV, family *Togaviridae*) was additionally assessed. Although some differences in infection levels were also observed between clones for these viruses, they were less pronounced than with SARS-CoV-2, and the susceptibility of the clones for these viruses did not correlate with the susceptibility to SARS-CoV-2 (Figure 1B), suggesting that clonal differences in SARS-CoV-2 infection probably resulted from differences in the expression of SARS-CoV-2-specific host factors.

Next, the expression levels of ACE2 and TMPRSS2, two host factors critically involved in SARS-CoV-2 entry, were analyzed by immunoblotting. ACE2 expression levels were highly variable from one clone to another (Figure 1C). For the clones with lower levels of ACE2, the susceptibility to SARS-CoV-2 infection correlated well (R^2^ = 0.965) with ACE2 expression (Figure 1D). In contrast, TMPRSS2 was not detected in any clone or in the parental population (data not shown), as expected based on previous reports (Hoffmann et al., 2020). These data indicate that the heterogeneity of the Vero-81 cell population with respect to susceptibility to SARS-CoV-2 infection arises from variable expression levels of the SARS-CoV-2 receptor ACE2 in individual cells.

### Construction and evaluation of a fluorescent reporter system

**Figure 2:**
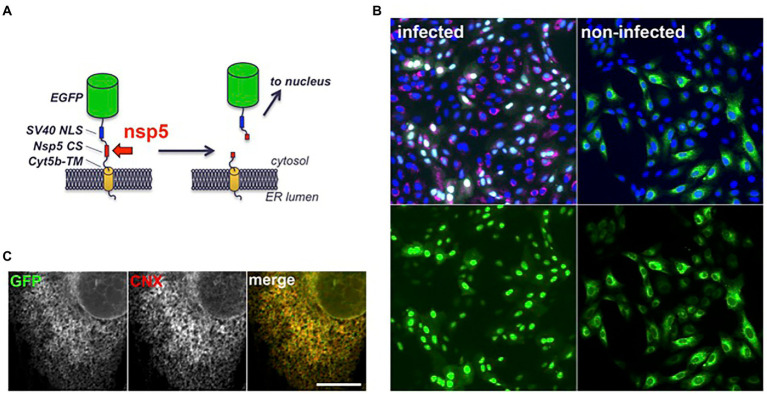
Structure and intracellular localization of a fluorescent SARS-CoV-2 reporter. **(A)** Scheme of the fluorescent reporter. EGFP: enhanced GFP (green), NLS: nuclear localization signal (blue), CS: cleavage site (red), cyt5b TM: cytochrome 5b transmembrane domain (yellow). **(B)** Vero-81-derived clone 6 cells expressing the fluorescent reporter (green) were infected with SARS-CoV-2 (left panels) or left non-infected (right panels). Cells were fixed and processed for immunofluorescent detection of dsRNA (red) and DAPI staining (blue) at 16 hpi. Same fields are shown with combined fluorescent signals (top) or with GFP signal only (bottom). **(C)** Confocal image of an non-infected clone 6 cell expressing the fluorescent reporter (GFP) immunolabeled with an antibody to calnexin (CNX). Individual channels are shown in grayscales for a better display and combined images in colors in the right panel. Bar, 10 μm.

To facilitate the quantification of SARS-CoV-2 infection in cell culture, a fluorescent reporter probe was constructed by inserting a substrate of the nsp5 protease between a GFP and a membrane anchor (Figure 2A). The sequence of the nsp4/nsp5 autocleavage site (SAVLQSGF) was chosen as the cleavage site of the construct, and the cytochrome 5b C-terminal domain as a membrane anchor to localize the construct at the cytosolic side of the ER membrane. In that way, the substrate should be accessible to nsp5 and its cleavage should release GFP from its membrane anchor and generate a soluble product. To spatially separate the original substrate and the cleaved product in fluorescence microscopy, a nuclear localization signal was fused to GFP, in order to display non-infected cells with a cytoplasmic/ER-like fluorescence and infected cells with a nuclear pattern. Indeed, Vero-81-derived clone 6 cells transfected with a plasmid encoding this fluorescent reporter displayed a cytoplasmic fluorescence (Figure 2B). Its localization at the ER membrane was confirmed by colocalization with calnexin, an integral membrane protein of the ER (Figure 2C). As predicted, the fluorescent signal shifted from the cytoplasm to nuclei upon infection (Figure 2B, green signal). To confirm that cells exhibiting nuclear staining were indeed infected, they were labeled with an antibody directed against dsRNA, an intermediate in RNA replication specifically labeling infected cells (Figure 2B, red signal). All cells with nuclear GFP were positive for dsRNA. In contrast, less than 2% of dsRNA-negative cells had a fluorescent nucleus. These results confirm that the presence of GFP in the nucleus is an accurate marker of SARS-CoV-2 infection. However, only ~83% of dsRNA-positive cells displayed a fluorescent nucleus at 16 hpi, suggesting that the quantification of infection using the fluorescent probe was slightly less sensitive than with dsRNA immunofluorescence.

**Figure 3:**
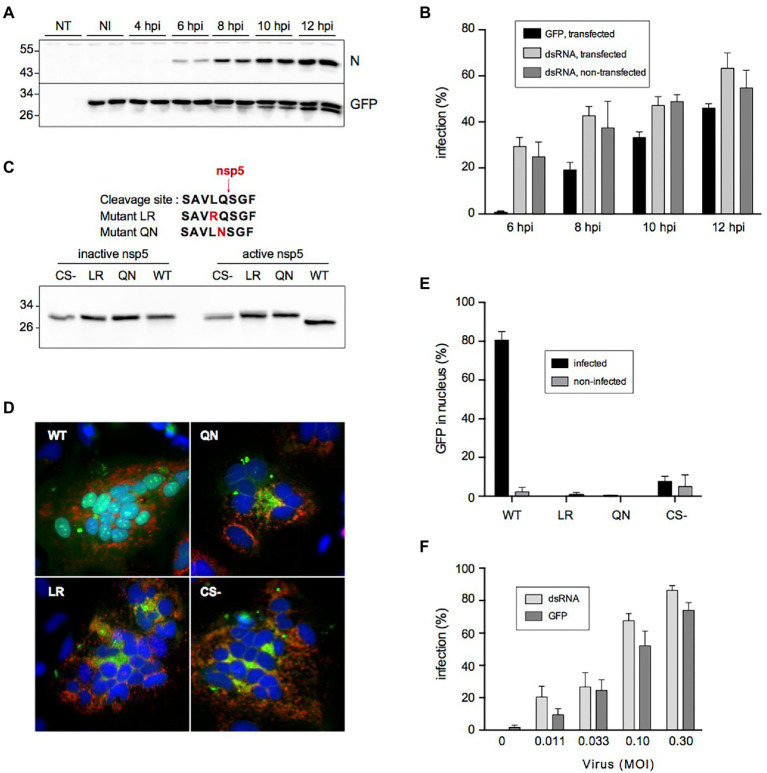
Cleavage of the fluorescent reporter in SARS-CoV-2-infected cells. **(A)** Kinetics of cleavage analyzed by immunoblotting. NT: non-transfected, NI: non-infected, N: nucleocapsid, GFP: fluorescent reporter detected with an antibody to GFP. **(B)** Kinetics of infection quantified by nuclear translocation (GFP) in cells transfected with the fluorescent reporter and by dsRNA immunostaining in transfected or non-transfected cells (mean ± SD of 6 replicates). **(C)** Impact of mutating Leu and Gln residues on the cleavage by SARS-CoV-2 nsp5. The amino acid sequences of the mutant cleavage sites are shown on top. HeLa cells were co-transfected with indicated constructs and an active or inactive version of nsp5 and analyzed by immunoblotting with an antibody to GFP. CS- is a construct lacking the whole cleavage site peptide. **(D)** Impact of cleavage site mutation on nuclear transfer of the reporter. HeLa cells were co-transfected with indicated constructs (green) and a plasmid expressing human ACE2 and infected with SARS-CoV-2. Cells were fixed and processed for immunofluorescent detection of dsRNA (red) and DAPI staining (blue) at 16 hpi. **(E)** Impact of cleavage site mutation on nuclear transfer of the reporter in Vero cells. Vero-81-derived clone 6 cells were transfected with indicated constructs and infected with SARS-CoV-2. Infected cells were defined by dsRNA immunostaining and the nuclear localization of GFP in infected and non-infected cells was quantified (mean ± SEM of 4 independent experiments). **(F)** Nuclear GFP staining as a function of virus input. The infection was measured by counting cells GFP staining (GFP) or by immunostaining to dsRNA (mean ± SD of 8 replicates).

Kinetics experiments revealed that probe cleavage was first detectable at 8 hpi and further increased over time (Figure 3A), whereas nucleocapsid protein expression was detectable from 6 hpi onward. This slight delay was also visible when the kinetics of infection were compared by counting infected cells with a green fluorescent nucleus on the one hand and by dsRNA immunofluorescence on the other hand, explaining why the latter was slightly more sensitive to quantify the number of infected cells (Figure 3B).

To confirm the nsp5-specific cleavage of the probe, the critically required Leu or Gln residues in the cleavage site of the fluorescent probe were changed into Arg or Asn residues, respectively (mutants LR and QN). Plasmids expressing either the wild-type probe, the LR and QN mutants, or a construct with no cleavage site at all (CS-) were co-transfected in HeLa cells with a plasmid expressing SARS-CoV-2 nsp5 or an inactive version of the protease. These experiments confirmed that the probe was indeed cleaved in nsp5 active conditions (Figure 3C). In contrast, none of the mutant constructs were cleaved, indicating that both mutations impaired nsp5-mediated cleavage. Then, these constructs and ACE2 were co-expressed in HeLa cells and these transiently transfected cells were infected with SARS-CoV-2. The infection was confirmed by the presence of syncytia and the detection of dsRNA (Figure 3D). All infected cells expressing the wild-type probe exhibited fluorescent nuclei, while infected cells expressing the mutant constructs displayed a cytoplasmic pattern of fluorescence, similar to the cells expressing the construct with no cleavage site (CS-). This shows that the presence of an nsp5 cleavage site is required for the nuclear translocation of the probe upon infection in ACE2-expressing Hela cells. When these constructs were expressed in Vero-81-derived clone 6 cells, none of the mutants displayed a nuclear fluorescence pattern upon SARS-CoV-2 infection, in contrast to the wild-type probe containing the nsp5 cleavage site, for which ~80% of infected cells exhibited fluorescent nuclei (Figure 3E). Taken together, these results confirm that the shift of fluorescence pattern upon infection requires the presence of a genuine nsp5 cleavage site in the probe and therefore strongly suggest that its cleavage is performed by nsp5 in infected cells.

Finally, to find out if the quantification of SARS-CoV-2 infection with the fluorescent reporter would indeed generate reliable results, clone 6 cells expressing the fluorescent reporter were inoculated with different doses of SARS-CoV-2 and the number of infected cells was quantified at 16 hpi both by counting the number of cells with a green fluorescent nucleus and with a ds-RNA-positive signal. Both quantification methods showed a virus dose-dependent increase in infection. Again, the percentage of infection was however slightly lower when assessed with the fluorescent reporter than with the anti-dsRNA immunofluorescence (Figure 3F). Nonetheless, as this reporter system allows a much faster quantification of virus infection, its value as a screening tool in a high-throughput format was further assessed.

### Application of the reporter system to a phenotypic high-throughput screening approach

**Figure 4:**
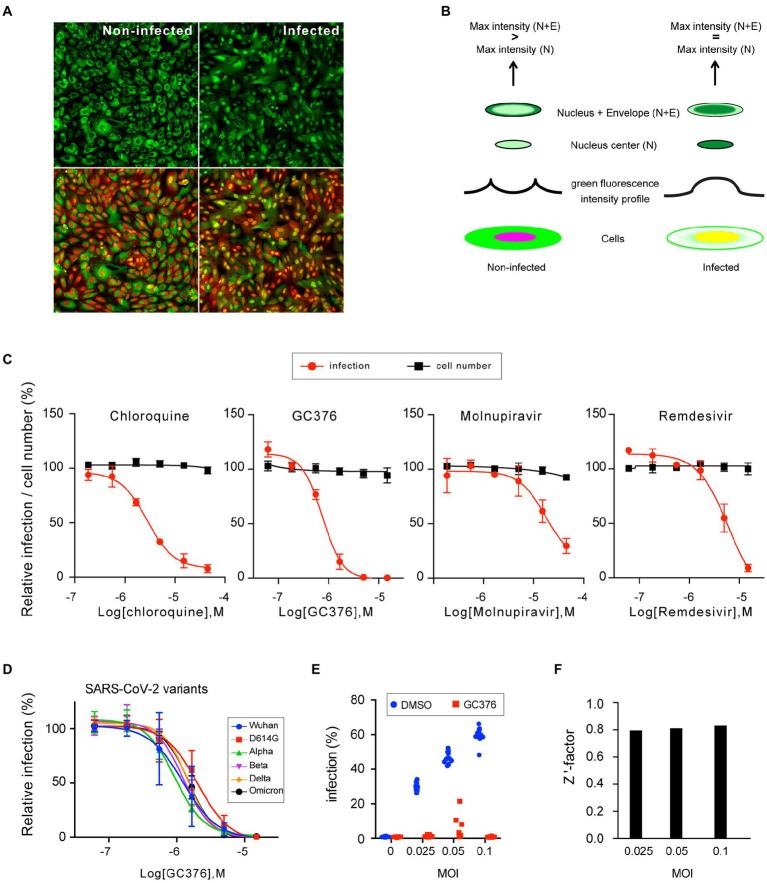
Characterization of the F1G-red reporter cell line in high-throughput format. **(A)** Images of individual fields of non-infected (left) and infected (right) F1G-red cells in 384-well plate format acquired using an InCell-6500 automated confocal microscope. The same fields are shown with GFP signal only (top) or with combined fluorescent signals (bottom). **(B)** Principle of automated quantification of SARS-CoV-2 infection. Cells are depicted with green and red signals, spatially separated in non-infected cells or combined in infected cells. The fluorescent intensity profile of GFP along an axis through the nucleus is schematically depicted above both cells. Above the intensity profiles, the pattern of green fluorescence in two areas, corresponding to the center of the nucleus (N) or to the nucleus and a ring of cytoplasm containing the nuclear envelope (N + E), are shown. Criteria for scoring infection versus non-infection are indicated on the top. **(C)** Antiviral dose–response experiments. F1G-red cells were infected with SARS-CoV-2 in the presence of indicated antivirals in 384-well plates and infection was measured at 16 hpi as explained in **(B)**. Relative infection rates (red line) and cell numbers (black line) normalized to DMSO controls are plotted (mean ± SEM of 3 (chloroquine and GC376) or 2 (remdesivir and molnupiravir) independent experiments). **(D)** GC376 dose–response experiments using the indicated SARS-CoV-2 variants of concern using F1G-red cells in 384-well plate format (mean ± SEM of 3 independent experiments). **(E)** F1G-red cells were infected with SARS-CoV-2 at different MOI in the presence of 10 μM GC376 or 0.1% DMSO in 384-well plates and infection was measured at 16 hpi as explained in **(B)**. Individual values corresponding to 12 independent wells are plotted for each condition. **(F)** A robust Z’ factor based on the median of each series of measurements shown in **(E)** was calculated for each MOI.

To apply this reporter system to a high content format, a clonal cell line stably expressing the fluorescent probe was generated. A clone, named F1G, stably expressing the probe was obtained by successive limiting dilutions. Additionally, to allow visualization of the nucleus without the need for addition of any exogenous reagent (such as DAPI or Hoechst), a red fluorescent protein with a nuclear localization signal (mCherry-NLS) was stably expressed in the F1G cells, finally yielding a cell line named F1G-red, expressing the green fluorescent probe in the cytoplasm and a red fluorescent marker in the nucleus in non-infected conditions. As the green fluorescence shifts to the nucleus upon infection, both green and red signals co-localized in the nucleus in infected conditions (Figure 4A). Next, we designed a method to automatically quantify SARS-CoV-2 infection (supplementary data). For each cell, defined by its nucleus in the red channel, two fluorescence measurements in the green channel were performed, a first one in an area included within the nucleus (defined by 80% of the nucleus radius) and a second one including both the nucleus and a ring of cytoplasm around the nucleus containing the nuclear envelope (defined by 125% of the nucleus radius). By comparing the maximum fluorescence intensity between both areas, cells with the same signal can be scored as infected, whereas cells with a higher fluorescent signal in the ‘nucleus + envelope’ area are scored as non-infected (Figure 4B). In that way, cells can be infected and the green and red fluorescence signals are directly recorded and automatically quantified in living cells.

Using this automated method, we assessed the possibility of generating reliable dose–response curves with a small series of known antivirals, including two nucleotide analogs, remdesivir and molnupiravir (Jeon et al., 2020; Sheahan et al., 2020; Vangeel et al., 2022), the coronavirus nsp5 protease inhibitor GC376 (Kim et al., 2012; Fu et al., 2020; Ma et al., 2020; Vuong et al., 2020), and chloroquine, which is an endocytosis-mediated entry inhibitor in Vero cells (Hoffmann et al., 2020). As shown in Figure 4C, dose–response curves were obtained for each of the tested antivirals, with EC_50_ values of 2.8 μM for chloroquine, 0.75 μM for GC376, 6.1 μM for remdesivir, and 17 μM for molnupiravir (Figure 4C, red curves), which are in the ranges of previous reports on the antiviral effect of these drugs in Vero cells (Hoffmann et al., 2020; Jeon et al., 2020; Wang et al., 2020), except for molnupiravir that was previously reported with EC_50_ values in sub/low-μM range (Sheahan et al., 2020; Vangeel et al., 2022). The reason for this lower activity remains unknown, but it has to be stated that all experiments were performed in absence of a P-glycoprotein efflux inhibitor, and hence that EC_50_ values might have been overestimated here (Hoffman et al., 2020; Vangeel et al., 2022). Quantification of the mCherry-based total cell numbers showed an absence of clear cytotoxicity at the tested concentrations (Figure 4C, black curves).

To assess the susceptibility to different SARS-CoV-2 variants, dose–response experiments were performed with GC376 using 6 SARS-CoV-2 variants, since it has been shown that M^pro^ mutations in these variants remain sensitive to GC376 (Greasley et al., 2022; Sacco et al., 2022; Ullrich et al., 2022). As expected, F1G-red cells were responsive to all variants (Figure 4D), and the EC_50_ values for GC376 calculated for all these variants were in the same range (from 0.94 μM for the alpha variant to 2.0 μM for the D614G variant). Again, these EC_50_ values might have been overestimated due the absence of a P-glycoprotein efflux inhibitor in these experiments.

Finally, to assess the potential of this assay for high-throughput screenings (HTS), the Z’-factor was tested as an assay quality indicator. Infections of F1G-red cells were performed at different MOI in the presence of DMSO or GC376 at a concentration of about 10xEC_50_ (Figure 4E). A robust version of the Z’-factor, commonly used in HTS data analysis, was applied to sets of 12 independent measures of each condition for each MOI (Figure 4F). This revealed a value of about 0.8 for this assay, whatever the MOI (ranging from 0.795 at MOI = 0.025 to 0.833 at MOI = 0.1), which is rated as an “excellent assay” according to (Zhang et al., 1999).

## Discussion

In December 2019, the world was introduced to a novel respiratory disease, COVID-19, caused by a newly emerged human coronavirus, later named SARS-CoV-2. After the emergence of SARS-CoV-1 in 2002 and MERS-CoV in 2012, SARS-CoV-2 was the third human coronavirus with high pathogenic potential. In sharp contrast to the 2 previous ones, this new virus surprised friend and foe with its rapid spread among the human population, resulting in a worldwide pandemic from March 2020 onward. Since then, massive amount of work has been done to identify anti-SARS-CoV-2 molecules that together with the available vaccines should help in controlling this pandemic, few of which have been approved for use in patients so far. However, the rapid need to screen massive amounts of molecules also highlighted the lack of a good cell-based model that allowed for a fast, effortless, and automated read-out of the SARS-CoV-2 infection, and that required minimal handlings to ensure maximum safety in the BSL-3 environment.

The present paper reports the construction and characterization of a new reporter cell line for quantifying SARS-CoV-2 infection in living cells and shows that it can be used for automated dose–response calculations of antiviral compounds using a high content format. The main advantage of this reporter system is that it allows for a fast quantification of infection (before the onset of CPE) with minimally required handlings, as it does not require any additional reagent to measure infection. Indeed, there is no need to add any DNA probe, such as DAPI or Hoechst, to define the position of nuclei before recording images, and the detection of infected cells does not require adding any substrate or antibody, unlike reporter systems based on luciferase activity or immunostaining (Xie et al., 2020; Bakowski et al., 2021; Tanaka et al., 2022). This makes it easily amenable to large sets of measures in a 384-well plate format and safer for the operator in a BSL-3 environment. In this model, cells were defined by the co-occurrence of green and red fluorescence, and the infection status was scored according to the pattern of green fluorescence, which allowed us to develop a simple ‘infect-and-read’ protocol for quantifying SARS-CoV-2 infection.

Another advantage of this system is that it relies on *in situ* detection of nsp5 proteolytic activity, which is highly specific to SARS-CoV(−2) infection, in contrast to other cell-based protocols relying on indirect and/or late impacts of infection, such as the measure of syncytia formation (Buchrieser et al., 2020) or of cell mortality (Riva et al., 2020; Belouzard et al., 2022; Chiu et al., 2022). Moreover, it allows for quantification of infection with any of the yet arisen variants of SARS-CoV-2. This could be of great interest to quickly confirm the activity of antiviral compounds against any new variant. An approach similar to ours has already been reported for other viruses, including HCV, dengue virus, and SARS-CoV-2 (Jones et al., 2010; Pahmeier et al., 2021), and used to follow the kinetics of infection in living cells by video microscopy. This further highlights the flexibility of such fluorescent reporter systems, which might be of great value to study other (corona)viruses.

In the present paper, it was decided to use Vero-81 cells as a basis to make the reporter cell line, although this is not a physiologically relevant model for SARS-CoV-2 infection, which typically occurs in respiratory epithelial cells. The rationale behind the use of this African green monkey kidney cell line is dual, because these cells are not only highly susceptible to SARS-CoV-2 and can be easily cultured and cloned, but also because infection in these cells occurs without syncytium formation, which severely complicates software-based automated counting of infected cells. Therefore, as the main aim of the present study was not to provide a physiologically relevant cell model but rather an easy-to-use cell-based assay that allows for a first selection of potential antiviral molecules when massive amounts of molecules need to be screened, the Vero-81-based cell model was chosen. Another limitation of this model is that it cannot be used to study antivirals acting on the more physiologically relevant TMPRSS2-mediated entry pathway, which is absent in Vero-81 cells, and hence that this might lead to false-positive hits targeting the endosomal pathway, such as chloroquine. However, this can be easily solved by ectopic expression of TMPRSS2 in the F1Gred cells. Moreover, the high efflux of some chemicals in Vero cells (Hoffman et al., 2020) can potentially yield overestimated EC_50_ values, compared to IC_50_ values measured in other assays. Therefore, antiviral screenings should ideally be performed in the presence of a P-glycoprotein efflux inhibitor in this cell model. Although the fast read-out after inoculation is a great advantage to speed up screenings, it makes this model especially suited to screen for antivirals that execute their effect at early stages of the replication cycle. Nonetheless, we believe that this cell line provides an ideal model for a fast, safe, and automated initial screening of large batches of molecules, after which the antiviral activity of the selected groups of molecules should be further characterized in other cell models, such as TMPRSS2-expressing cells, and more relevant respiratory-tract derived cell models, before proceeding into animal models.

## Data availability statement

The raw data supporting the conclusions of this article will be made available by the authors, without undue reservation.

## Author contributions

JD, SB, and YR: conceptualization. LD, NC, EH, AD, ML, CC, SB, and YR: investigation. LD and YR: writing–original draft preparation. LD, EH, CC, JD, SB, and YR: writing–review and editing. JD and SB: funding acquisition. All authors contributed to the article and approved the submitted version.

## Funding

This work was supported by the Centre National de la Recherche Scientifique (CNRS: COVID and ViroCrib programs). The platform used in this work was supported by the European Union (ERC-STG INTRACELLTB grant 260901), the ANR (ANR-10-EQPX-04-01), the “Fonds Européen de Développement Régional” (Feder) (12001407 [D-AL] EquipEx ImagInEx BioMed), CPER-CTRL (Centre Transdisciplinaire de Recherche sur la Longévité), and the Région Nord-Pas-de-Calais (convention 12000080).

## Conflict of interest

The authors declare that the research was conducted in the absence of any commercial or financial relationships that could be construed as a potential conflict of interest.

## Publisher’s note

All claims expressed in this article are solely those of the authors and do not necessarily represent those of their affiliated organizations, or those of the publisher, the editors and the reviewers. Any product that may be evaluated in this article, or claim that may be made by its manufacturer, is not guaranteed or endorsed by the publisher.
