# Supplementary material for: A reporter cell line for the automated quantification of SARS-CoV-2 infection in living cells
Source: Front Microbiol. 2022 Sep 29;13:1031204. doi: 10.3389/fmicb.2022.1031204 (PMC9558224; doi:10.3389/fmicb.2022.1031204)
Supplement: Supplementary file 1 [file Data_Sheet_1.PDF]

Supplementary table 1. Multiparametric script used in Columbus to determine the percentage of infected cells and the total number of cells.

Analysis Sequence

Input Image	Input		
	**Flatfield Correction :** None<br>Brightfield Correction<br>**Stack Processing :** Individual Planes<br>**Min. Global Binning :** Dynamic		

Filter Image	Input	Method	Output
	**Channel :** Orange	**Method :** Sliding Parabola<br>Curvature : 2	Output Image : Sliding Parabola

Find Nuclei	Input	Method	Output
	**Channel :** Sliding Parabola<br>**ROI :** None	**Method :** B<br>Common Threshold : 0.4<br>Area : > 30 µm²<br>Splitting Coefficient : 7.0<br>Individual Threshold : 0.4<br>Contrast : > 0.1	Output Population : Nuclei

Select Population	Input	Method	Output
	**Population :** Nuclei	**Method :** Common Filters<br>Remove Border Objects<br>Region : Nucleus	Output Population : Nuclei Selected

Calculate Morphology Properties	Input	Method	Output
	**Population :** Nuclei Selected<br>**Region :** Nucleus	**Method :** Standard<br>Area<br>Roundness	Property Prefix : Nucleus

Step	Input	Method	Output
**Select Population (2)**	**Population :** Nuclei Selected	**Method :** Filter by Property<br>Nucleus Area [µm²] : > 100<br>Nucleus Roundness : > 0.85<br>Nucleus Area [µm²] : < 400<br>Boolean Operations : F1 and F2 and F3	Output Population : Noyaux
**Select Cell Region**	**Population :** Noyaux	**Method :** Resize Region [%]<br>Region Type : Nucleus Region<br>Outer Border : 20 %<br>Inner Border : 100 %	Output Region : Nucleus Region
**Select Cell Region (2)**	**Population :** Noyaux	**Method :** Resize Region [%]<br>Region Type : Ring Region<br>Outer Border : -20 %<br>Inner Border : 100 %	Output Region : Ext Region
**Calculate Intensity Properties**	**Channel :** Green<br>**Population :** Noyaux<br>**Region :** Nucleus Region	**Method :** Standard<br>Mean<br>Median<br>Maximum<br>Minimum<br>Contrast	Property Prefix : Intensity Nucleus Region Green
**Calculate Intensity Properties (2)**	**Channel :** Green<br>**Population :** Noyaux<br>**Region :** Ext Region	**Method :** Standard<br>Mean<br>Median<br>Maximum	Property Prefix : Intensity Ext Region Green

		Minimum	

Select Population (3)	Input	Method	Output
	**Population :** Noyaux	**Method :** Filter by Property<br>Intensity Nucleus Region Green Maximum : > 2000<br>Intensity Ext Region Green Maximum : > 2000<br>Boolean Operations : F1 or F2	Output Population : Noyaux Selected

Calculate Properties	Input	Method	Output
	**Population :** Noyaux Selected	**Method :** By Formula<br>Formula : A/B<br>Variable A : Intensity Nucleus Region Green Maximum<br>Variable B : Intensity Ext Region Green Maximum	Output Property : ratio

Select Population (4)	Input	Method	Output
	**Population :** Noyaux Selected	**Method :** Filter by Property<br>ratio : == 1	Output Population : infected

Select Population (5)	Input	Method	Output
	**Population :** Noyaux Selected	**Method :** Filter by Property<br>ratio : < 1	Output Population : non inf

Define Results	Results
	**Method :** List of Outputs<br>**Population : Noyaux Selected**<br>Number of Objects<br><br>**Population : Noyaux**<br>Number of Objects<br><br>**Population : Nuclei Selected**

Number of Objects

Population : infected
Number of Objects

Population : non inf
Number of Objects

Method : Formula Output
Formula : a/b*100
Population Type : Objects
Variable a : infected - Number of Objects
Variable b : Noyaux Selected - Number of Objects
Output Name : % infection

Object Results
Population : Noyaux Selected : None
Population : Noyaux : None
Population : Nuclei Selected : None
Population : Nuclei : None
Population : infected : None
Population : non inf : None
